# New therapeutic directions in type II diabetes and its complications: mitochondrial dynamics

**DOI:** 10.3389/fendo.2023.1230168

**Published:** 2023-08-21

**Authors:** Shengnan Wang, Haiyang Zhao, Suxian Lin, Yang Lv, Yue Lin, Yinai Liu, Renyi Peng, Huanzhi Jin

**Affiliations:** ^1^ Department of Rheumatology and Immunology, The Third Affiliated Hospital of Shanghai University, Wenzhou No.3 Clinical Institute Affiliated to Wenzhou Medical University, Wenzhou People’s Hospital, Wenzhou, China; ^2^ Institute of Life Sciences & Biomedicine Collaborative Innovation Center of Zhejiang, College of Life and Environmental Science, Wenzhou University, Wenzhou, China; ^3^ General Practitioner, The Third Affiliated Hospital of Shanghai University, Wenzhou No.3 Clinical Institute Affiliated to Wenzhou Medical University, Wenzhou People’s Hospital, Wenzhou, China

**Keywords:** mitochondrial dynamics, mitochondrial fusion, mitochondrial fission, type II diabetes, diabetic complications

## Abstract

As important organelles of energetic and metabolism, changes in the dynamic state of mitochondria affect the homeostasis of cellular metabolism. Mitochondrial dynamics include mitochondrial fusion and mitochondrial fission. The former is coordinated by mitofusin-1 (Mfn1), mitofusin-2 (Mfn2), and optic atrophy 1 (Opa1), and the latter is mediated by dynamin related protein 1 (Drp1), mitochondrial fission 1 (Fis1) and mitochondrial fission factor (MFF). Mitochondrial fusion and fission are generally in dynamic balance and this balance is important to preserve the proper mitochondrial morphology, function and distribution. Diabetic conditions lead to disturbances in mitochondrial dynamics, which in return causes a series of abnormalities in metabolism, including decreased bioenergy production, excessive production of reactive oxygen species (ROS), defective mitophagy and apoptosis, which are ultimately closely linked to multiple chronic complications of diabetes. Multiple researches have shown that the incidence of diabetic complications is connected with increased mitochondrial fission, for example, there is an excessive mitochondrial fission and impaired mitochondrial fusion in diabetic cardiomyocytes, and that the development of cardiac dysfunction induced by diabetes can be attenuated by inhibiting mitochondrial fission. Therefore, targeting the restoration of mitochondrial dynamics would be a promising therapeutic target within type II diabetes (T2D) and its complications. The molecular approaches to mitochondrial dynamics, their impairment in the context of T2D and its complications, and pharmacological approaches targeting mitochondrial dynamics are discussed in this review and promise benefits for the therapy of T2D and its comorbidities.

## 1 Introduction

Mitochondria represent one of the major resources of reactive oxygen species (ROS) and the main part of ATP production (1, 2). In the persistent hyperglycemic state of diabetes, mitochondria will increase the output of ROS, which leads to oxidative stress (OS) and tissue damage (3–5). Hyperglycemia can lead to a relative increase in ROS in three ways (causing respiratory chain electron transport blockage, burst production of ROS, and damage to the antioxidant system), mediating mitochondrial OS, damaging mitochondria and causing mitochondrial dysfunction (4, 6, 7). The normal function of mitochondria relies in their efficient quality of control with a highly plasticity of their dynamic structure, that makes them continually changeable through fusion and fission processes, namely mitochondrial dynamics (8, 9).

Mitochondrial fusion consists of outer membrane (OMM) and inner membrane (IMM) fusion, with OMM fusion are modulated by mitofusin-1 (Mfn1) and mitofusin-2 (Mfn2), whereas IMM fusion are modulated by optic atrophy 1 (Opa1) (10, 11). Dynamin related protein 1 (Drp1) is a crucial protein in mediating mitochondrial division, and other proteins involved in fission include mitochondrial fission factor (MFF) and mitochondrial fission 1 (Fis1) (11, 12). Regulatory mitochondrial dynamics is a complicated process in which mitochondria are coregulated by the above GTPases to maintain the homeostasis of their fission and fusion, which is fundamental to cellular functions including mitochondrial DNA distribution, mitochondrial functions, cell survival and signaling (8, 13, 14). Alterations in this balance may result in OS, mitochondrial impairment and metabolic modification, finally contributing to the occurrence of mitochondrial-related diseases (8, 15, 16).

Mitochondrial dynamics are markedly changed in type II diabetes (T2D) patients and are involved in the progression of insulin resistance, and not only that, mitochondrial dynamics are also closely correlated with the evolution of diabetic complications (6, 17–19). Therefore, it is significant to investigate the association between alterations in mitochondrial dynamics and T2D and its complications, and this article reviews existing research progress in this area.

## 2 Mitochondrial dynamics

Mitochondrial dynamics means that the dynamic equilibrium in which mitochondria are in a constant fusion and fission process. The mitochondrial fusion and fission processes are synergistic, highly conserved processes, and the two are generally in dynamic equilibrium, a balance that is essential for sustaining the proper mitochondrial morphology, distribution and function (8). Both abnormal fusion and fission will affect the normal function of mitochondria, for example, abnormal fission will result in small and broken mitochondrial morphology and abnormal fusion will result in extended mitochondria (6, 20).

### 2.1 Mitochondrial fusion

The morphology of mitochondria can be changed by mitochondrial fusion, which refers to the phenomenon of two adjoining mitochondria merging their contents into one mitochondria with fibrous extensions and a network structure, and is present in all cells with mitochondria (10, 11, 21). Mitochondrial fusion is considered to be a key factor in maintaining a healthy mitochondrial network. The underlying principle is to achieve dilution of toxic substances in mitochondria and repolarization of membrane potential through fusion for the purpose of maintaining genetic and biochemical homogeneity (14). This function results in the selective fusion of healthy and less healthy mitochondria, while depolarized unhealthy mitochondria are rapidly become targets for decomposition (22, 23). Thus, in contrast to the active selection of fusion partners by mitochondria, the cytoplasmic environment is perhaps the pivotal determinant of fusion events, and a change in any one substance would affect this process (11, 14).

**Figure 1:**
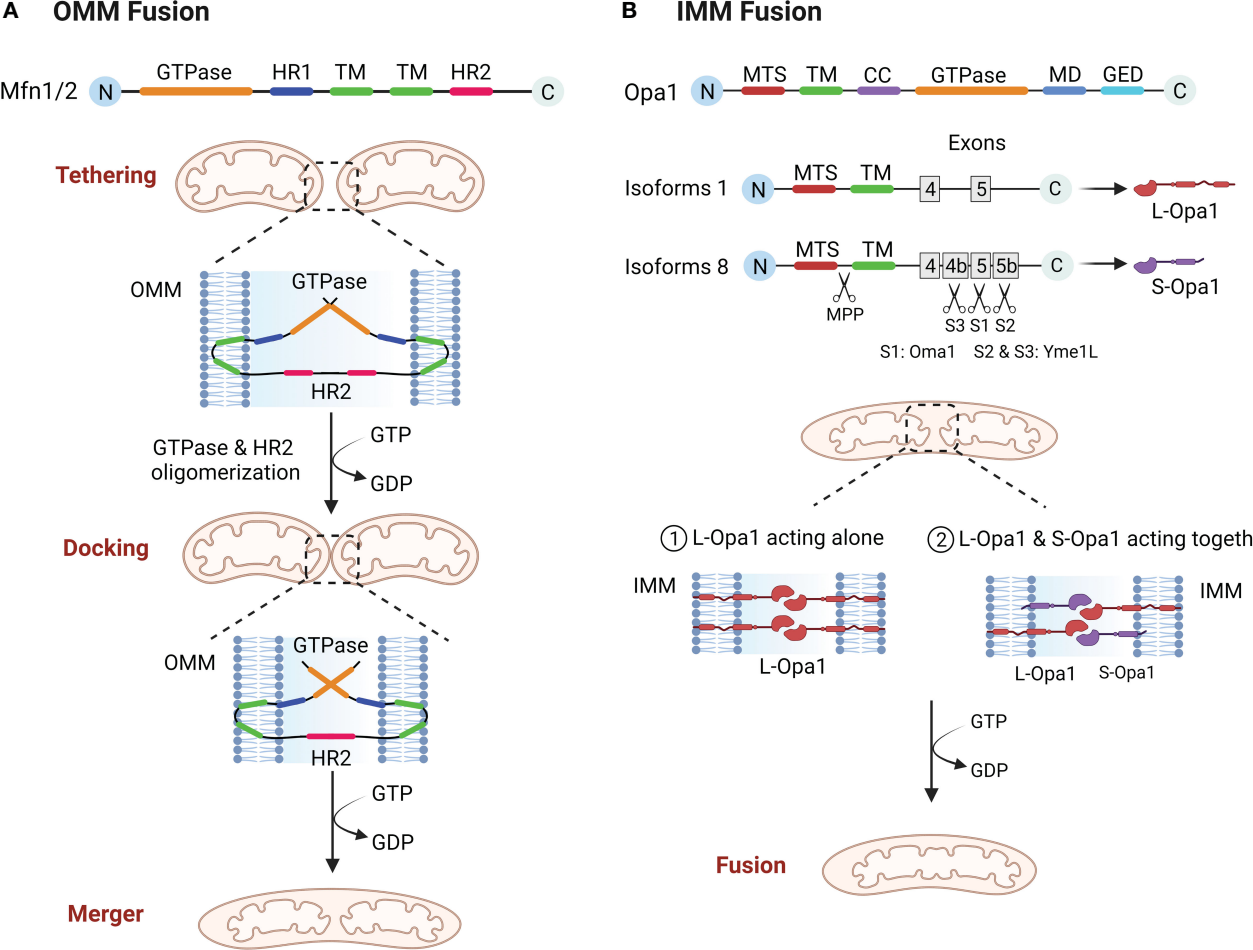
**(A)** OMM fusion. Mfn1 and Mfn2 tether mitochondria through oligomerization of HR2 and GTPase domain, and as GTP is hydrolyzed, mitochondria move closer together, eventually leading to OMM fusion. **(B)** IMM fusion. Opa1 needs to be hydrolyzed to L-Opa1 and S-Opa1 to promote IMM fusion. The figure shows two isoforms of Opa1, the mitochondrial targeting sequence (MTS) is first excised by MPP, and because isoform 1 lacks exons 4b and 5b it will not be cut, resulting in L-Opa1. While isoform 8 is cleaved in the S1 site by Oma1 and in the S2 and S3 sites by Yme1L, giving rise to S-Opa1. L-Opa1 then mediates IMM fusion either alone or in conjunction with S-Opa1.

The structure of the Mfn molecule mediating outer mitochondrial membrane (OMM) fusion contains an GTPase domain, two hydrophobic heptapeptide repeat helix regions (HR1 and HR2), with two transmembrane domains (TM) (24, 25). The GTPase domain is essential for fusion activity and the TM is required for OMM insertion (24, 26). Mfn mediated OMM involves three steps, tethering, docking and merger (10). The two mitochondria must be in close contact to begin fusion, and once close contact is established, the OMM of the two mitochondria form trans-homologous or heterologous complexes *via* the HR2 and GTPase domains of Mfn, mediating OMM fusion (26–29). Mfn undergoes local and global conformational changes during GTP hydrolysis and forms a dimer through the GTPase domain to complete the OMM tether. As the cycle of GTP hydrolysis process continues, the mitochondria are continuously drawn closer together, eventually achieving OMM fusion (**Figure 1A**) (10, 29). Functionally, Mfn1 and Mfn2 are considered essential for mitochondrial fusion, and both have not only a degree of similarity as well as substantial differences (30). The tether efficiency of Mfn1 on mitochondrial membrane is higher than Mfn2, and the GTPase activity of purified Mfn1 is eight times more than the one of Mfn2 (29). In addition, defects in either Mfn1 or Mfn2 will result in abnormal mitochondrial morphology and reduced fusion rates. In Mfn2 deficient fibroblasts mitochondria appeared spherical or round with swelling, whereas Mfn1 deficient mitochondria were significantly more brokenness (31).

After the OMM fusion, the IMM fusion must be followed up quickly and efficiently to completion (27). The structural composition of OPA1 is an mitochondrial targeting sequence (MTS), a subsequent TM, a coiled coil domain (CC), a highly conserved GTPase domain, an middle domain (MD) and a GTPase effector domain (GED) (10, 32). IMM fusions are more complex than OMM fusions in that the *Opa1* gene coding has eight distinct isoforms and three cleavage sites (S1, S2, S3) and requires partial protein hydrolysis into two forms, L-Opa1 and S-Opa1, to successfully complete the fusion reaction (33, 34). Some of these researches suggest that L-Opa1 is integral to IMM and promotes IMM fusion, whereas S-Opa1 does not mediate fusion (35). However, Song and Ge et al. demonstrated that IMM fusion requires the joint collaboration of S-Opa1 and L-Opa1 (36, 37). Therefore, the exact mechanism of IMM fusion will have to be confirmed by further research. Analogous with Mfn proteins, Opa1 also constitutes oligomeric structures that drive IMM fusion through conformational changes driven by GTP hydrolysis (**Figure 1B**) (38). In addition, Opa1 is involved in cristae shaping and electron tomography has shown that Opa1 modulates the morphology of mitochondrial cristae, especially during apoptosis (39).

### 2.2 Mitochondrial fission

**Figure 2:**
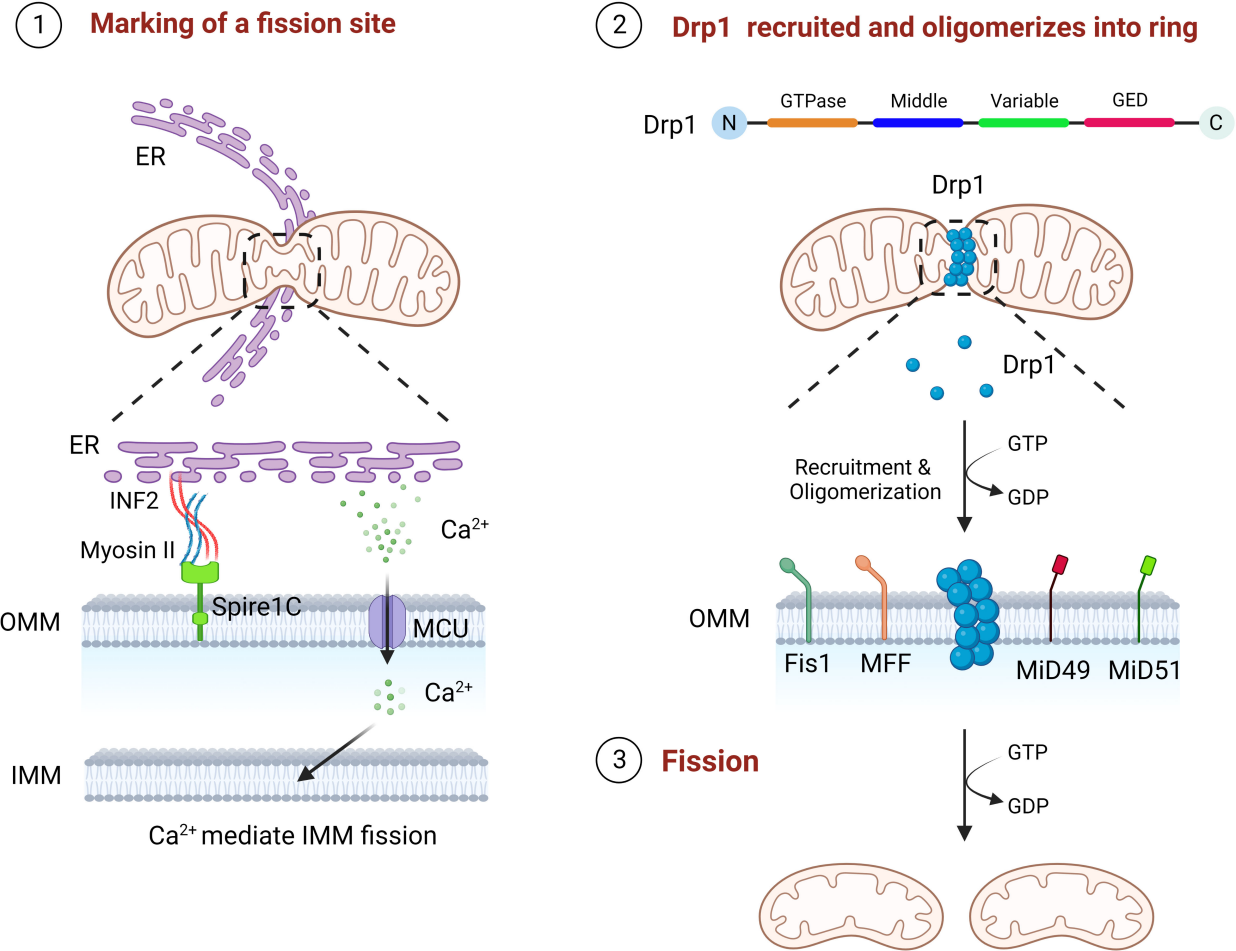
The three steps of mitochondrial fission. First, ER contacts mitochondria, polymerizes with Spire1C on mitochondria to form a fission site through INF2 and myosin II, and releases Ca^2+^ into mitochondria to mediate IMM fission. Drp1 is then recruited by Fis1, MFF, MiD49 and MiD50 to the mitochondrial fission site and oligomerizes into a loop. Finally, through GTP hydrolysis, Drp1 continuously contracts mitochondria until fission.

Mitochondria can divide to produce one or more daughter mitochondria, mainly mediated by Drp1, which has four different structural domains, including the GTPase domain, MD, variable domain and GED (11, 12). Drp1 in the cytoplasm need to be translocate to the mitochondria carry out its divisive function (40). The mitochondrial fission process undergoes three critical phases, marking of the fission site, oligomerization of Drp1 at the fission site and assembly into a helical superstructure, and contraction of the Drp1 and severing of the mitochondria (14). The preliminary step of mitochondrial fission is the initial contraction of the OMM, which is produced by the endoplasmic reticulum (ER) and actin filaments (41, 42). ER contacts mitochondria to form mitochondria-associated ER membranes (MAMs) to mediate the forming of mitochondrial fission sites, reducing the average mitochondrial diameter through the polymerization of ER-associated inverted formin 2 (INF2), myosin II, and Spire1C at the MAMs, which contributes to the formation of a Drp1 oligomerization loop at the fission site (41, 43, 44). Then there is the Drp1 recruitment, which is mainly accounted for by a class of small molecule proteins that are mosaic on the OMM (45). These include Fis1, MFF, 49 kDa and 51 kDa of mitochondrial dynamics protein (MiD49 and MiD51), the deletion of each of these molecules leads to significant stretching of the mitochondria (45). Drp1 is translocated to the fission site of the OMM in the cytoplasm and oligomerized, then assembles to a helical superstructure (46). Finally, the mitochondrial conformation is altered by hydrolysis of GTP, which continues to enhance mitochondrial contraction until two mitochondria are created (**Figure 2**) (11, 47). Following mitochondrial fission, Drp1 can be returned to the cytoplasm for reuse (11).

In addition, INF2 mediated actin polymerization facilitates the Ca^2+^ liberation from the ER at the MAMs, followed by Ca^2+^ passes through the mitochondrial calcium unidirectional transporter protein (MCU) into the mitochondria leading to IMM contraction (48). As the S637 residue of Drp1 can be dephosphorylated by Ca^2+^ dependent calcineurin, persistent elevated cytoplasmic Ca^2+^ levels further promotes Drp1 aggregation in mitochondria, suggesting that IMM fission is independent of, and precedes, OMM fission (48–50). More interestingly, S-Opa1 produced by Opa1 hydrolysis has been found to act in mitochondrial fission, where stress induced OMA1 hydrolyzes all of Opa1 into a short isomers and S-Opa1 is able to partly co-position with the MAMs, thereby inhibiting fusion and triggering mitochondrial breakage (51).

## 3 The role of mitochondrial dynamics balance

Mitochondrial fusion and fission are engaged directly or indirectly in mitochondrial maintenance, bioenergy production and cell death, which is essential in the development of diabetes mellitus and its complications (12, 13).

### 3.1 Mitochondrial dynamics influence oxidative phosphorylation processes

**Figure 3:**
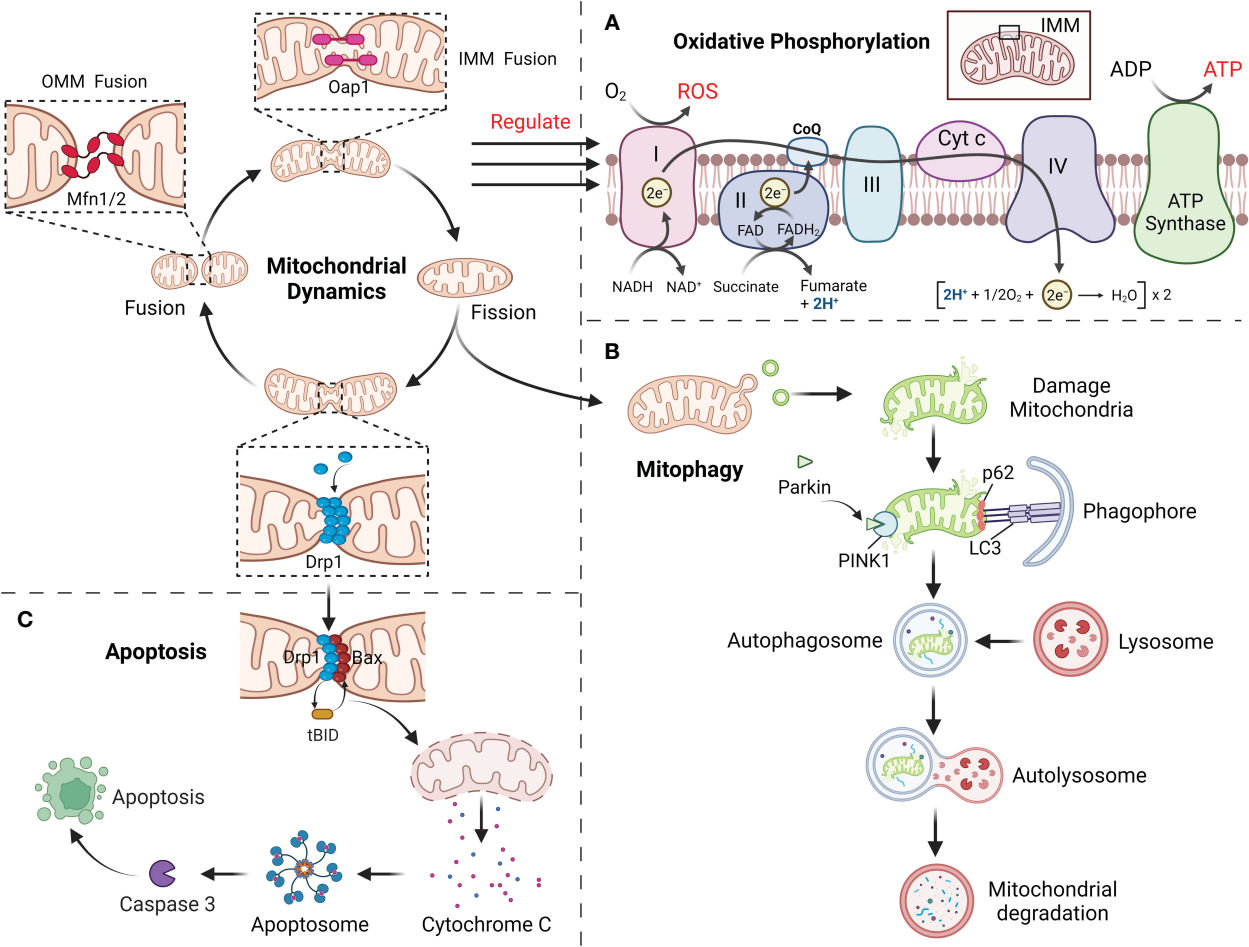
**(A)** Mitochondrial dynamics regulate the OXPHOS process, with fusion promoting OXPHOS and fission inhibiting it. **(B)** The damaged mitochondria first divide the damaged site through mitochondrial fission, and then degraded by mitophagy. **(C)** Mitochondrial fission mediates apoptosis through the release of Cyt c.

ATP produced by mitochondria through respiration and oxidative phosphorylation (OXPHOS) is the main source of energy in cell and along with the production of ROS (52, 53). Impaired OXPHOS leads to overproduction of ROS and gradually inhibit or deplete the body’s antioxidant system, eventually lead to the disorder of the body’s redox balance and result in OS and tissue damage (6, 54). As a vital mechanism for the homeostatic modulation of mitochondria, mitochondrial dynamics can be participated in the modification of OXPHOS processes (5). It is suggest that mitochondrial morphology may determine the level of intracellular ATP supply and the mode of respiratory metabolism (9, 55). Mitochondria in the fused state are rich in internal cristae structure, with close connected with the electron transport chain and high efficiency of OXPHOS. This is attributed to the fact that Opa1 and Mfn1/2 dependent mitochondrial fusion preserves mtDNA mass to maintain the electronic respiratory chain (ETC) complex and overall OXPHOS capacity (56, 57). In addition, Mfn2 could bind to a glycolysis rate-limiting enzyme (pyruvate kinase) to enhance mitochondrial OXPHOS and attenuate glycolysis (58). When the level of Drp1 mediated mitochondrial fission is elevated, OXPHOS capacity is reduced and the bioenergetic state is shifted to glycolysis, with a concomitant decrease in mitochondrial membrane potential and an increase in ROS production. By blocking Drp1 phosphorylation, mitochondrial fusion can be redirected and OXPHOS can be activated (56, 57). However, excessive mitochondrial fusion equally damages OXPHOS (57). Thus, coordinated mitochondrial dynamics homeostasis is essential for maintaining OXPHOS function and induces mitochondrial OS (**Figure 3A**).

### 3.2 Mitochondrial dynamics affect mitophagy

Mitochondria can maintain their homeostatic function by selectively degrading excess or damaged mitochondria within the cell, and this mechanism is called mitophagy (23, 59). Before mitophagy, the damaged mitochondria require Drp1 for mitochondrial fragments of appropriate size by mitochondrial fission (59). Moreover, Mfn1/2 and Opa1 in damaged mitochondria are degraded to prevent greater damage caused by the fusion of damaged mitochondria with healthy ones (23, 60). Mitophagy mainly involves PTEN-induced putative kinase 1 (PINK1) and the E3 ubiquitin ligase Parkin (59). When mitochondria have been injured, PINK1 accumulates in the OMM *via* outer membrane translocases, activating and recruiting Parkin, followed by ubiquitination of the proteins VDAC1 and Mfn1/2 in the mitochondrial outer by Parkin (61, 62). Subsequently, p62 accumulates on mitochondria, combines with ubiquitinated mitochondria together with LC3 and is phagocytosed by autophagosomes, which merge with lysosomes to form autophagic lysosomes that degrade the contained mitochondria (**Figure 3B**) (63). In addition, several mitochondrial LC3 receptors have been described that are independent of PINK1 in ubiquitin-independent autophagy, including BNIP3, Nix, FUNDC1 and BCL2L13, which include LC3-interacting regions that bind directly to LC3 and assemble damaged mitochondria to the autophagosome (64, 65). Mitochondrial dynamics are inextricably linked to mitophagy. FUNDC1 interacts with Drp1 and Opa1, and under normal conditions FUNDC1 can bind to Opa1 to promote mitochondrial fusion, whereas under stress conditions FUNDC1 detaches from Opa1, enhancing the recruitment of Drp1 by FUNDC1 at the MAMs and promoting mitochondrial fission as well as mitochondrial autophagy (66, 67). When mitophagy is impaired, mitochondrial dynamics will likewise be affected. PHB2 has been proved to be required for PINK1-mediated mitophagy, and PHB2 deletion causes selective absence of the long isoform of Opa1, which results in abnormal cristae morphogenesis and mitochondrial fragmentation (68, 69). Abolishing mitophagy induced by BNIP3L and FUNDC1 during cardiac progenitor cell differentiation will also result in consistent mitochondrial fission and donut-like formation of damaged mitochondria (70). This shows a strong association with mitochondrial dynamics and mitophagy.

### 3.3 Mitochondrial dynamics affect apoptosis

Mitochondria as a vital regulator of apoptosis, which play a vital role in the progression of apoptosis by liberating cytochrome c (Cyt c) and other pro-apoptotic factors (71, 72). Drp1, an important mitochondrial fission protein, not only affects mitochondrial morphology but also participates in the regulation of apoptosis (73). Mitochondrial fission itself does not lead to apoptosis, but mainly pro-apoptotic proteins (such as Bax) from the Bcl-2 family members are co-expressed with Drp1 at the fission site responsible for Cyt c release, an important early event in caspase 3 activation that ultimately induces apoptosis (74, 75). During apoptosis, recruitment of Drp1 is enhanced and Drp1 stimulates tBid-triggered Bax oligomerization by promoting hemifusion of cardiolipin-containing membranes and then initiates apoptosis by releasing Cyt c through a membrane hemifusion intermediate formed during mitochondrial fission (**Figure 3C**) (74, 75). Even in the absence of apoptotic triggers, Drp1 can directly regulate the Bax pore to affect the permeability activity of Bax to promote apoptosis. This is because the N-terminal structural domain of Bax interacts specifically and directly with Drp1 to form a homodimeric complex until cell death, and this interaction is enhanced during apoptosis (74).Downregulation of *Drp1* caused by RNAi not only retards mitochondrial fission, but also inhibits Cyt c release and cell death (76). Fis1 was also involved in the regulation of apoptosis, and downregulation of *Fis1* inhibited apoptosis to a significantly greater extent than downregulation of *Drp1* (77). However, during cell differentiation, reduced levels of mitophagy induced by decreased *Drp1* activity promote the early stages of apoptosis, whereas its overexpression prevents apoptosis, which provides another important link between apoptosis and mitochondrial dynamics (78). Furthermore, silencing of *Mfn1* or *Mfn2* will lead to increased mitochondrial fragmentation and sensitivity to apoptotic stimuli (79). Opa1 blocks the tBid-induced increase in Cyt c release from the cristae into the membrane gap, and Opa1 deficiency will also induce mitochondrial cristae abnormalities as well as spontaneous cell apoptosis (79). Restoration of mitochondrial fusion delays Bax activation and Cyt c release and reduces cell damage/apoptosis.

## 4 The function of mitochondrial dynamics in T2D and its complications

### 4.1 The effect of altered mitochondrial dynamics on the development of T2D

**Figure 4:**
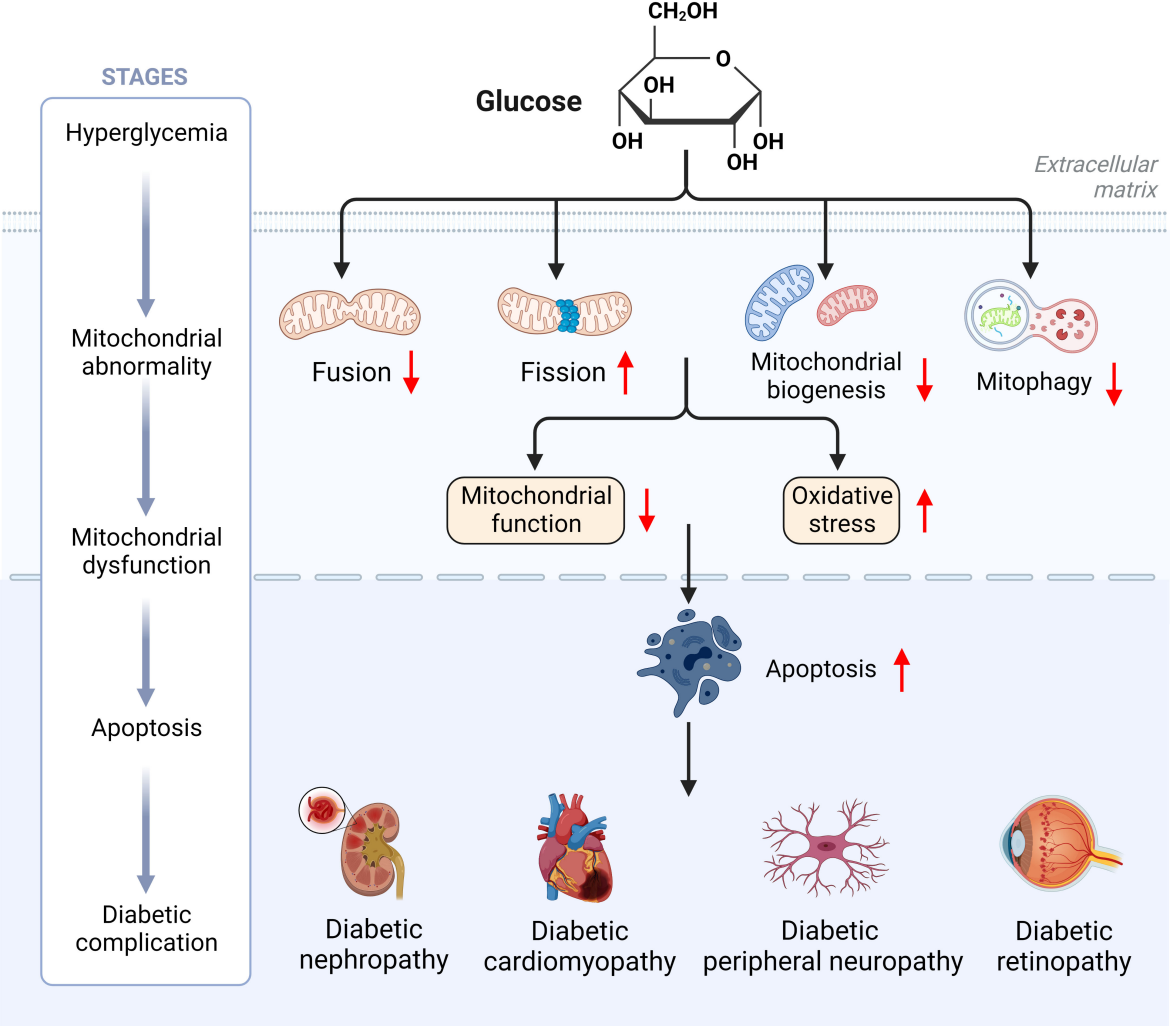
Hyperglycemia induces mitochondrial dysfunction ultimately leading to diabetic complications.

T2D is mainly distinguished by mitochondrial dysfunction, increased ROS content and decreased ATP levels (6). In T2D, hyperglycemia induces excessive mitochondria division and fragmentation in different cell types, with excessive production of ROS, reduced mitochondrial fusion and increased mitochondrial fission eventually resulting in mitochondrial dysfunction (80, 81). Under normal physiological conditions, damaged mitochondria are eliminated by mitophagy before they lead to excessive ROS production (81). In contrast, hyperglycemia in T2D inhibits the expression of mitophagy-related genes (*NIZ, PINK1, Parkin*), leading to impaired mitophagy (82). Reduced ATP levels due to mitochondrial dysfunction can be compensated by mitochondrial biogenesis, however, downregulation of a gene involved in mitochondrial biogenesis (*PGC-1α*) was observed in T2D (83). Abnormal mitochondrial biogenesis leads to energy dysregulation and accelerates ROS production, thereby aggravating the pathological pathway of diabetes development (84). Thus, abnormal mitochondrial dynamics induced by hyperglycemia may be the pathogenesis and common basis for the genesis of chronic complications of diabetes mellitus (**Figure 4**).

### 4.2 The effect of altered mitochondrial dynamics on the development of diabetic nephropathy (DN)

The kidney is a highly metabolic, energy-consuming and mitochondria-rich organ second only to the myocardium. In the diabetic state, the impairment of mitochondrial dynamics resulting in reduced mitochondrial quantity and capacity, and the accumulation of ROS are also considered to be a critical link the pathogenesis and evolution of DN, leading to a large number of cytokines and inflammatory mediators are released and downstream signaling pathways such as protein kinase C are activated, which further destroys the intrinsic cells of the kidney (podocytes, glomerular endothelial cells, thylakoid cells and tubular epithelial cells) and promotes the genesis and evolution of DN (15, 85–87).

Studies of renal cells in the diabetic setting showed altered renal mitochondrial dynamics, with increased mitochondrial *Drp1* expression and decreased *Mfn2* expression, suggesting that mitochondria tend to undergo fission and that the fusion process is inhibited (88). Disturbances in renal mitochondrial dynamics lead to histological and renal parameter abnormalities, *Drp1* overexpression can lead to membrane matrix expansion, basement membrane thickening, podocyte damage and albuminuria (89). The associated proteins participating in mitochondrial fission and fusion are altered during the various stages of DN, and genetic modification of them can alleviate the symptoms of DN (88, 90). For example, DN mice specifically overexpressing *Mfn2* or deletion of *Drp1* can significantly improve kidney injury and mitochondrial ROS accumulation (91). Ultrastructural analysis showed that diabetic mice specifically deficient in Drp1 had reduced proteinuria and significantly improved membrane matrix expansion and foot cell morphology as well as mitochondrial structure compared with wild-type diabetic mice (91). Additionally, in Rho-associated kinase 1 (ROCK1) transgenic mice, it was observed that hyperglycemia phosphorylated Drp1 serine residues *via* ROCK1, thereby promoting the recruitment of Drp1 into mitochondria and ultimately inducing mitochondrial fission, while knockdown of ROCK1 reduced glomerular apoptosis and mitochondrial ROS production, decreased proteinuria and reduced podocyte shedding (92). Administration of Mdivi1, a pharmacological inhibitor of Drp1, significantly attenuated mitochondrial fission in mice and salvaged critical pathological characteristics of DN (93).

Asiatic acid was recently found to reduce tubular injury in DN by decreasing the expression of *Drp1* and increasing the expression of *Mfn1/2 via* the Nrf-2 pathway (94). Existing studies have identified a variety of drugs that can modulate mitochondrial dynamics to alleviate the symptoms of DN, as shown in **Table 1**. In addition to alleviating the symptoms of DN, the modulation of mitochondrial dynamics may further exacerbate the damage of DN. When diabetic rats are administered Crocodile Oil, it will further increase *Drp1* expression and decrease *Mfn2* levels (95). Crocodile Oil use exacerbates diabetic kidney injury with a significant increase in mitochondrial ROS production and a decrease in mitochondrial membrane potential (MMP) compared to diabetic rats that do not receive Crocodile Oil (101). Therefore, by targeting and regulating mitochondrial fusion division-associated protein expression will influence the progression of DN. In addition, mitophagy, which is closely related to mitochondrial dynamics, is also expected to serve as a new target for the treatment of DN. PACS-2 promotes mitophagy and plays an important role in ameliorating tubular injury in diabetes (95).

**Table 1:** Drugs targeting mitochondrial dynamics in the treatment of DN.

Medicine	Signal pathway	Mitochondrial dynamic change	Curative effect	Ref
HIF-1α	HO-1	*Mfn1*, *Mfn2* expression ↑, *Drp1*, *Fis1* expression ↓	Renal tubular injury ↓	(87)
Empagliflozin	AMPK/ SP1/PGAM5	Inhibition of *Drp1* dephosphorylation in S637 and translocation	Renal tubular injury ↓	(15)
PACS-2	–	*Mfn2* expression ↑, *Drp1*, *Fis1* expression ↓	Renal tubular injury ↓	(95)
Formononetin	Sirt1/PGC-1α	*Mfn2* expression ↑, *Drp1*, *Fis1* expression ↓	Albuminuria and renal tubular cell apoptosis ↓	(96)
Berberine	–	*Drp1*, *Fis1*, *MFF*, *Mid49*, *Mid51* expression ↓	Podocyte injury ↓, basement membrane thickening ↓, mesangial dilatation and glomerulosclerosis ↓	(89)
DUSP1	DUSP1- JNK-MFF	*MFF* expression ↓	Glomerular apoptosis and renal fibrosis ↓	(97)
Melatonin	–	*Mfn2*, *Opa1* expression ↑, *Drp1* expression ↓	Proteinuria ↓and creatinine clearance ↑	(98)
Sitagliptin	SDF3α/CXCR1	*Mfn2*, *Opa1* expression ↑, *Drp1* expression ↓	Glomerular and tubulointerstitial injury ↓	(99)
Asiatic acid	Nrf-2	*Mfn1*, *Mfn2* expression ↑, *Drp1* expression ↓	Renal function and tubular injury ↓	(94)
Mitochondria-Targeted Peptide SS31	–	*Mfn1* expression ↑, *Drp1* expression ↓	Renal tubulointerstitial injury ↓, serum creatinine and microalbuminuria levels ↓	(93)
Polydatin	–	*Drp1* expression ↓	Podocyte injury ↓	(100)

Note, ↑ mean rise, ↓ mean decrease.

### 4.3 The effect of altered mitochondrial dynamics on the development of diabetic cardiomyopathy (DCM)

DCM is a nonischemic and nonhypertensive cardiomyopathy caused by diabetes-related metabolic disorders and represents one of the common diabetic complications (102). Deficiency of myocardial energy is highly associated with the development and evolution of various cardiac pathologies (103). Under normal physiological conditions, mitochondrial OXPHOS accounts for the vast majority of cardiac ATP requirements; however, in the presence with insulin resistance, myocardial use of insulin-stimulated glucose uptake and glucose utilization is reduced and fatty acid uptake and oxidation rates are increased, and this altered substrate preference plays an essential role in the pathophysiology of DCM (103–105). When fatty acid intake in the heart increases, it can cause mitochondrial structural remodeling, significantly reduce the minimum diameter, and regulate Opa1 and Drp1 post-translational modifications, thereby promoting mitochondrial fission (106).

DCM is initially marked by myocardial fibrosis, left ventricular hypertrophy and diastolic dysfunction, which later manifests as systolic dysfunction and eventually clinical heart failure (102, 104, 107). Heart failure is distinguished by altered redox regulation of the myocardium, mainly in the form of OS, and increased ROS have been described in animal models of heart failure (107–109). We have previously described the association between mitochondrial dynamics and OS, and alterations in mitochondrial dynamics are observed in all pathological changes in DCM, as shown in **Table 2**. Disturbed mitochondrial dynamics in cardiomyocytes, followed by mitochondrial OXPHOS and mitochondrial respiratory chain dysfunction, lead to reduced ATP production and excessive ROS production, ultimately leading to cardiomyocyte death (112). Hu’s study indicated that the reduced expression of Mfn2 in DCM could be partly attributed to the result of reduced expression of peroxisome proliferator-activated receptor α (PPARα) (112). Mfn2 deficiency not only leads to impaired glucose tolerance, the progression of hyperinsulinemia and insulin resistance, but also affects the normal development of cardiac muscle cells (117). Mitophagy has been shown to be critical in protecting cardiac function during DCM, and Mfn2 may have a pivotal role in cardiac autophagy by facilitating fusion between autophagic vesicles and lysosomes (118, 119). Parkin-mediated mitophagy protects against high-fat diet-induced cardiac invasion of hypertrophy, lipid accumulation and diastolic dysfunction in the heart, and when mitophagy is impaired, mitochondrial dysfunction and lipid accumulation are induced, thereby exacerbating DCM (118).

**Table 2:** Mitochondrial dynamics and morphology under DCM in different species.

Species	Functional disorder	Mitochondrial dynamic change	Mitochondrial morphology	Ref
Mouse	Diastolic dysfunction	*Drp1* phosphorylation ↑ and *Mfn2* expression ↓	Mitochondrial fragmentation	(110)
	Heart failure	*L-Opa1* missed	Mitochondrial fragmentation	(111)
	Cardiac hypertrophy and fibrosis	*Mfn2* expression ↓	–	(112)
Rat	Cardiomyocyte hypertrophy	*Drp1* expression ↑	Mitochondrial fragmentation	(113)
	Myocardial fibrosis	*Drp1* expression ↑, *Mfn1, Mfn2* expression ↓	–	(114)
Human	Myocardial contractility decreased	*Mfn1* expression ↓	Mitochondrial network fragmentation	(115)
	Heart failure	*Opa1* expression ↓	Mitochondria are small and broken	(116)

Note, ↑ mean rise, ↓ mean decrease.

Calcineurin (CaN) is an important regulator of cardiac hypertrophy and heart failure, and CaN is activated in response to increased calcium concentrations in cells (120, 121). High glucose stimulation induces upregulation of Orai1 (Ca^2+^ release-activated calcium channel protein 1) expression, and Orai1 is able to mediate calcium inward flow to bind CaN and calmodulin (CaM). CaM then binds to CaN to form an active phosphatase that catalyzes subunit A (CnA) and phosphorylates- extracellular signal-regulated kinase (p-ERK1/2) as targets to suppress Drp1 phosphorylation at S637 and induce Drp1 phosphorylation at S616, respectively, facilitating mitochondrial fission and speeding up cardiac hypertrophy induced by high glucose (113, 122). Suppression of the Orai1-Ca^2+^-CnA or ERK-Drp1 prevents high glucose induced cardiomyocyte hypertrophy (113). Excessive mitochondrial fission in DCM patients and administration of the mitochondrial fusion promoter M1 dramatically enhanced mitochondrial fusion and increased *Opa1* expression levels in diabetic hearts, improving myocardial fibrosis and OS in cardiomyocytes (123). Melatonin supplementation also inhibited Drp1 mediated fission further reducing OS and promoting cardiomyocyte survival during hyperglycemic stress (124). Furthermore, by regulating the upstream factors of mitochondrial dynamics might be a new pathway for the treatment of DCM. Paeonol and Brain natriuretic peptide promotes Opa1-mediated mitochondrial fusion in the DCM to maintain cardiac mitochondrial function through activation of the CK2α-Stat3 pathway and PKG-STAT1 pathway, respectively (125, 126). Nicotinamide riboside promotes Mfn2-mediated mitochondrial fusion *via* the SIRT2-PGC1α-PPARα pathway, reduces diabetes-induced cardiomyocyte apoptosis, and prevents diabetes-induced cardiac insufficiency (127). Aldehyde dehydrogenase 2 (ALDH2) regulates mitochondrial fusion and fission through the PI3K/AKT/mTOR pathway in DCM patients and attenuates ischemia and reperfusion injury (128). Similarly, secreted frizzled related-protein 2 (SFRP2) and andrographolide have been successively reported to alleviate DCM in recent years by regulating the balance of mitochondrial dynamics (129, 130). Therefore, maintaining the myocardial mitochondrial fusion and fission balance is key to maintaining myocardial mitochondrial function, and targeting mitochondrial dynamics is a target for potentially intervening in metabolic disorder-related myocardial diseases such as diabetic cardiomyopathy and obesity cardiomyopathy.

### 4.4 The effect of altered mitochondrial dynamics on diabetic peripheral neuropathy (DPN)

DPN is a frequent neurological complication of diabetes, manifested by sensory, motor or autonomic disorders (131). Hyperglycemia and hyperlipidemia in diabetic patients facilitate the pathogenesis of neuropathy (132). The increase in long-chain saturated fatty acids due to dyslipidemia will induce mitochondrial depolarization and impair axonal mitochondrial transport, thereby impairing the mitochondrial dynamics of sensory neurons and ultimately ATP loss and neuronal apoptosis (132, 133). The excess glucose associated with hyperglycemia triggers nutrient overload in neurons, overloading glycolysis and the tricarboxylic acid cycle, leading to impaired OXPHOS processes, depolarization of MPP, inhibition of the rate of electron transfer and reduced ATP production (132, 134). These alterations in neuronal bioenergetics are accompanied by an increase in intracellular OS (135). To compensate for this reduction in bioenergy, neurons increase mitochondrial mass in response to OS through the division of existing mitochondria (136). However, excess glucose enhances the expression of the pro-apoptotic proteins Bim and Bax as well as Drp1, activating and localizing and thus promoting pro-apoptotic fission in mitochondria (137).

Painful diabetic neuropathy (PDN) is a clinical manifestation of DPN that includes small fiber degeneration and neuropathic pain, manifested by pathological pain and overexcitation of dorsal root ganglion (DRG) neurons (138). In a high fat diet induced PDN mouse model, mitochondria in DRG injurious neurons have a broken morphology after 2 weeks, prior to episodes of mechanically abnormal pain and small fiber degeneration (139). This indicates that overly fission and fragmented mitochondrial morphology may be the basis for PDN axon degeneration. DRG injured neurons also show elevated calcium levels, which may be the result of mitochondrial dysfunction (139). Decreased ATP levels reduce Na^+^-K^+^ ATPase function, elevate intracellular Na^+^, and reverse Na^+^-Ca^2+^ exchange, leading to elevated calcium levels in neuronal cell axons and mediating increased calcium-dependent mitochondrial fission (140). Persistent elevation of Ca^2+^ has been shown to be a critical ingredient of the signaling pathway causing axonal degeneration in the central and peripheral nervous system (141, 142). Through the specific removal of mitochondrial calcium transporters to prevent calcium from entering the mitochondria, normal mitochondrial morphology can be restored and axonal degeneration can be prevented (139). Furthermore, sensory neurons with downregulated Mfn2 expression showed delayed transport of mitochondria to distal axons and reduced frequency of mitochondrial motility (143). This transport defect interrupts the correct positioning of mitochondria, disrupts axonal and synaptic dysfunction, and may eventually lead to axonal degeneration (144, 145).

Tang Luo Ning, a traditional Chinese compound prescription, significantly reduced the expression of mitochondrial fission protein in sciatic nerve Schwann cells, significantly increased the expression levels of Mfn1/2 and Opa1, and ameliorated nerve sheath disease in DPN rats (146). Isoliquiritigenin promotes mitochondrial biogenesis by mediating SIRT1 activation and regulates mitochondrial dynamics homeostasis to attenuate damage under diabetic neuropathy (147). Currently, studies targeting mitochondrial dynamics for the treatment of DPN are in their preliminary stages, it is evident that in diabetes, abnormalities in mitochondrial dynamics can lead to mitochondrial dysfunction resulting in neuronal damage and loss, and ultimately diabetic neuropathy. Therefore, by targeting mitochondrial dynamics possibly a promising approach for disease treatment in patients suffering from diabetic neuropathy.

### 4.5 The effect of altered mitochondrial dynamics on diabetic retinopathy (DR)

DR is one of the typical complications of diabetes and diabetic optic neurodegeneration is an early stage of DR pathogenesis and may be associated with the development of microvascular abnormalities (148, 149). Microvascular abnormalities within the retina include retinal vascular leakage and decellularized capillaries (149). In the pathogenesis of DR, accelerated capillary apoptosis by mitochondrial dysfunction is prior to the development of the histopathological features of DR (150, 151).

Mitochondrial disruption within diabetic retinal capillaries is partly related to the decreased expression of *Opa1* induced by diabetes (152). The research has demonstrated that *Opa1* expression were remarkably inhibited in the retinas of diabetic mice, where the proportion of *S-Opa1* to *L-Opa1* was also reduced (153). Opa1 reduction results in mitochondrial fragmentation through the development of mitochondrial swelling and local mitochondrial contraction (154). In addition, increased Bax expression and Cyt c release promote apoptosis of retinal endothelial cells (REC) and lead to increased acellular capillaries (AC) and pericyte loss (PL) which are features of DR pathogenesis (152, 153). These phenomena were more severe in diabetic mice with *Opa1* gene deletion (153).

Reduced expression of Mfn2 and overexpression of Drp1 and Fis1 were also found in the retinal choroidal system of animals and humans with DR, while Mfn1 levels remained unchanged (155, 156). Decreased Mfn2 expression may be due to DNA hypermethylation of its promoter in a hyperglycemic environment, leading to reduced binding of transcription factors and consequent inhibition of gene transcription (157). Regulation of Mfn2, Drp1 and Fis1 expression may protect mitochondrial homeostasis and suppress diabetic retinopathy. For example, reducing hyperglycemia-induced aberrant overexpression of *Drp1* and *Fis1* by combined siRNA approaches can effectively prevent mitochondrial breakage, improve mitochondrial respiration function and inhibit the apoptosis of RECs (156, 158, 159).

In summary, the both Mfn2 and Opa1 were downregulated under high glucose conditions is coincident with the upregulation of *Drp1* and *Fis1*. However, even if hyperglycemia is reversed to normal blood sugar levels, mitochondrial dynamics will still be impaired (160). Reduced fusion and increased fission of mitochondria eventually stimulate the discharge of Cyt c, inducing apoptosis in retinal cells like RECs and Müller cells (151). Thus, maintaining mitochondrial quality control and interfering with metabolic memory phenomena by directly regulating mitochondrial dynamics prevents further progression of DR (161). In recent years drugs that would prevent excessive mitochondrial fission have received much attention in the treatment of DR. Melatonin can guard the blood-retinal barrier by maintaining mitochondrial homeostasis. The mechanism is to upregulate the expression of genes linked to mitochondrial biogenesis (e.g., *PGC-1α*, *NRF2* and *PPAR-γ*) and downregulate the expression of genes linked to mitochondrial fission (e.g., *Drp1* and *Fis1*) (162). Drp1 can be de-SUMO-ized by SUMO-specific proteinase 3 (SENP3). Activation of de-SUMO-ized Drp1 disrupts mitochondrial dynamics, and increasing SUMO-ized Drp1 levels in retinal microvascular endothelial cells by inhibiting SENP3 expression reduces hyperglycemia-induced mitochondrial damage and apoptosis, thereby attenuating retinal permeability and increasing DR (163). Metformin prevents retinal ischaemia/reperfusion injury by increasing Mfn2 and Opa1-mediated mitochondrial fusion *via* AMPK (164). Current drugs and approaches that can be used to treat DR by targeting mitochondrial dynamics are shown in **Table 3**.

**Table 3:** Drugs and methods for targeting mitochondrial dynamics in the treatment of DR.

Medicine or treatment	Experimental subject	Mitochondrial dynamics target	Curative effect	Ref
Melatonin	Human retinal pigment epithelial cells	Mitochondrial fission-related genes expression ↓; mitochondrial biogenesis-related genes expression ↑	Apoptosis of retinal pigment epithelial cells ↓, protection of the blood-retinal barrier	(162)
SENP3-siRNA	Murine retinal microvascular endothelial cell	deSUMOylation of *Drp1* cause mitochondrial fission ↓	Blood-retinal barrier function and retinal tissue damage ↓	(163)
Tanshinone IIa	Bovine retinal endothelial cells	Mitochondrial overfitting ↓, mRNA levels of *Mfn1* and *Opa1* ↑	Oxidative stress and apoptosis of retinal endothelial cells ↓	(165)
Penicillamine	Human retinal pigment epithelial cell	*Mfn2* levels and restoration of mitochondrial biogenesis ↑	Endoplasmic reticulum stress and inflammation ↓, cell vitality ↑	(166)
TGR5	Human retinal vascular endothelial cells	Excessive mitochondrial fission mediated by the PKCδ/Drp1-HK2 ↓	Apoptosis of retinal vascular endothelial cells ↓	(167)
Mdivi-1	Human retinal vascular endothelial cells	Excessive mitochondrial fission mediated by the PKCδ/Drp1-HK2 ↓	Retinal vascular leakage and acellular capillaries ↓	(159)

Note, ↑ mean rise, ↓ mean decrease.

In addition, retinal cells could preserve themselves in a high glucose environment through mitophagy. Thioredoxin-interacting protein (TXNIP) is upregulated in a high glucose environment, inducing nitroso-modification of Drp1 and subsequently promoting TXNIP translocation to the mitochondria, mediating autophagy in a variety of pathways to maintain healthy mitochondria (168). The degree of hyperglycemia determines the level of mitochondrial autophagy; when the glucose concentration reaches 50 mM, mitophagy is inhibited, causing excessive mitochondrial fission and a tendency toward apoptosis (169, 170). Mitophagy has the potential to be a new therapeutic approach for DR.

## 5 Treatment of T2D and its complications through targeted modulation of mitochondrial dynamics

During the previous years, research on the exploitation of drugs or methods for mitochondrial fusion and fission has increased each year. One of the more thoroughly investigated drugs for excessive mitochondrial fission under hyperglycemic conditions is mdivi-1. In addition to repressing Drp1 activity in cells, mdivi-1 reduces OS and inflammation and increase insulin sensitivity in diabetic mice under insulin-resistant conditions (171, 172). However, when administered for more than 24 hours mdivi-1 will reduce the number of mitochondria and induce apoptosis (173). SGLT-2 inhibitors such as Dapagliflozin and Empagliflozin can reduce glucose by lowering the glucose uptake threshold and promoting the excretion of glucose from the urine (174). Not only that, SGLT-2 inhibitors also prevent mitochondrial swelling and enhance mitochondrial restoration and regeneration, and can improve mitochondrial dysfunction by inhibiting abnormal mitochondrial fission through AMPK (15, 175, 176). In addition, the use of SGLT-2 inhibitor drugs increases the risk of diabetic ketoacidosis (177).

In addition to drugs that broadly modulate mitochondrial dynamics, drugs applied to mitochondria in specific conditions have also been reported successively. For example, HIF-1α is a hypoxia-inducible factor that is expressed primarily in renal tubular cells and predisposes renal tubules to hypoxia (171, 172). It has been suggested that proximal renal tubule cells are the initiator and key therapy target of DN. HIF-1α can improve mitochondrial dynamics and limit mitochondria-dependent apoptosis in DN renal tubular cells through the HO-1 pathway (87, 172). This change may have occurred through the HO-1/CO pathway (87). The HO-1/CO pathway has been well characterized for its antioxidant and anti-inflammatory effects in *in vivo* and *in vitro* stress models, but its potential in regulating altered mitochondrial dynamics needs further investigation (178–180). While the potential benefits of these drugs with the ability to modulate mitochondrial dynamics are well documented, the adverse effects resulting from long-term use cannot be ignored (6).

In addition, MAMs is a new target for the treatment of diabetes and is involved in various physiological processes such as mitochondrial dynamics, mitochondrial autophagy, Ca^2+^ signaling and lipid metabolism (181, 182). In diabetes, hyperglycemia promotes excessive formation of MAMs in the body, leading to a range of mitochondrial dysfunctions (183). Fundc1 is a key molecule involved in MAMs formation, and by inhibiting its expression it can reduce the excessive formation of MAMs and effectively ameliorate diabetes and its complications, such as TRPV1 and SIRT3 (183–185). However, several other studies have contradicted this, indicating that mitochondrial dysfunction, apoptosis, and tissue damage in the diabetic setting are associated with disruption of MAMs integrity and a reduction in its formation (95, 186, 187). Further in-depth studies are still needed for the use of MAMs in diabetes and its complications.

## 6 Conclusion and future perspectives

**Figure 5:**
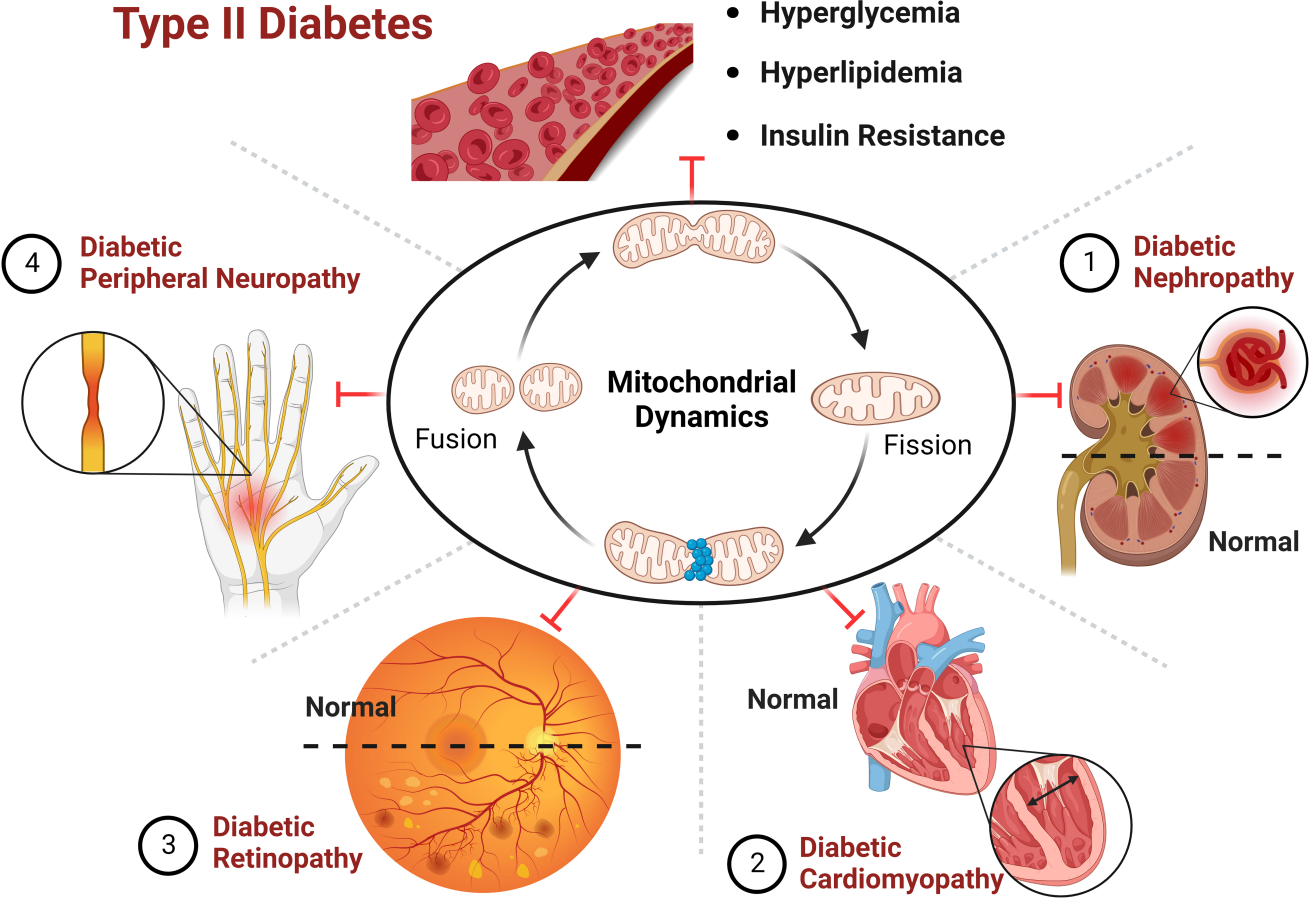
Improving the progression of diabetes and its complications by targeting mitochondrial dynamics.

Mitochondria are organelles with high dynamic changes in eukaryotic cells, and are one of the most important organelles in maintaining homeostasis in the body. It adapts to changes in the external environment, maintaining the function of tissues and organs through mitochondrial dynamics, and participating in various physiological procedures such as intracellular energy metabolism, autophagy and apoptosis, during which mis-regulation can lead to disease states, making their function vital to life. In the diabetic state, mitochondrial morphology is fragmented, and imbalance of mitochondrial dynamics causes disturbance in cellular energy metabolism and damage to pancreatic β-cells and peripheral tissues and organs, thus promoting the progression of diabetic complications. Targeting mitochondrial dynamic balance can effectively improve the progression of diabetes and its complications (**Figure 5**). The contribution of mitochondrial dynamics in chronic complications such as DN and DCM is gradually being explored, but a holistic picture is lacking and the specific molecular mechanisms and pathways of action are yet to be elucidated. There have been significant breakthroughs in recent years in the therapy of type II diabetes and its complications through targeted modulation of mitochondrial dynamics. The development of new therapeutic drugs based on the idea of adjusting the homeostasis of mitochondrial dynamics is expected to bring new light to the treatment of T2D and its comorbidities.

## Author contributions

SW and HZ contributed equally. SW and HZ, Investigation, data curation, writing - original draft, writing - review & editing. SL, YaL and YuL, Software, Supervision, Validation. YiL, RP and HJ, Conceptualization, Writing- Reviewing and Editing. All authors contributed to the article and approved the submitted version.
