# Supplementary figures and images for: New therapeutic directions in type II diabetes and its complications: mitochondrial dynamics
Source: Front Endocrinol (Lausanne). 2023 Aug 21;14:1230168. doi: 10.3389/fendo.2023.1230168 (PMC10475949; doi:10.3389/fendo.2023.1230168)

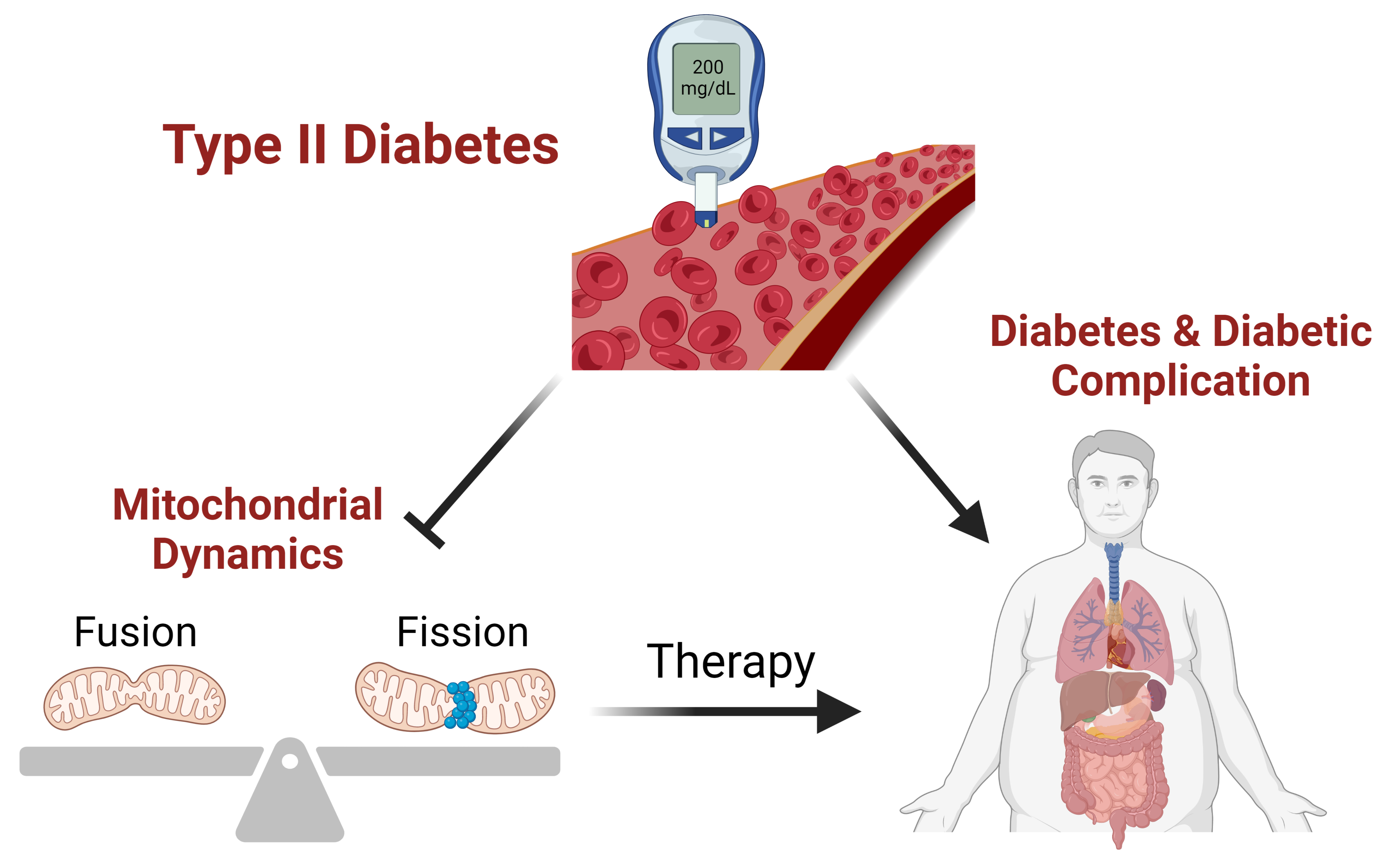

Supplement: Supplementary file 1 [file Image_1.jpeg]
